# Impact of Caffeine on Alzheimer’s Disease Pathogenesis—Protective or Risk Factor?

**DOI:** 10.3390/life12030330

**Published:** 2022-02-22

**Authors:** Thomas Gabriel Schreiner, Bogdan Ovidiu Popescu

**Affiliations:** 1 Faculty of Medicine, University of Medicine and Pharmacy “Carol Davila”, 050474 Bucharest, Romania; bogdan.popescu@umfcd.ro; 2 Department of Neurology, University of Medicine and Pharmacy “Gr. T. Popa”, 700115 Iasi, Romania; 3 Department of Electrical Measurements and Materials, Faculty of Electrical Engineering and Information Technology, Gheorghe Asachi Technical University of Iasi, 21-23 Professor Dimitrie Mangeron Blvd., 700050 Iasi, Romania; 4 Neurology Department, Colentina Clinical Hospital, 020125 Bucharest, Romania; 5 Laboratory of Cell Biology, Neurosciences and Experimental Myology, ‘Victor Babes’ National Institute of Pathology, 050096 Bucharest, Romania

**Keywords:** coffee, caffeine, Alzheimer’s disease, risk factors, protective factors, computational model

## Abstract

Alzheimer’s disease (AD), the most common dementia worldwide, remains without an effective treatment to this day despite intensive research conducted during the last decades. In this context, researchers have turned their attention towards the prevention of this pathology, focusing on early detection and better control of the most important risk factors, concomitantly with trying to find potentially protective factors that may delay the onset of AD. From the multitude of factors studied, coffee (especially its main component, caffeine) is a current interesting research topic, taking into consideration the contradictory results of recent years’ studies. On the one hand, much of the evidence from fundamental research suggests the potentially protective trait of caffeine in AD, while other data mainly from human studies lean toward no correlation or even suggesting that caffeine is a veritable risk factor for dementia. Given the methodological heterogeneity of the studies, this review aims to bring new evidence regarding this topic and to try to clearly establish a correlation between the two entities. Thus, in the first part, the authors make a clear distinction between the effects of coffee and the effects of caffeine in AD, presenting a rich basis of clinical trials on both animal models and the human subject. Subsequently, the main pathophysiological mechanisms that would explain the action of caffeine in the etiopathogenesis of AD are reviewed. Finally, the role of computational models is presented, having beneficial impact on both better understanding of the disease mechanism and the development of new therapeutic approaches for AD prevention.

## 1. Introduction

Coffee consumption is a daily habit for a significant percentage of the world’s population [1], caffeine being the most used psycho-stimulant in the western world [2,3]. With variations related to the daily consumed amount of coffee (low, moderate, and high consumption), the type of coffee beans used (the majority containing 10.0–12.0 mg of caffeine/g of coffee bean), and the method of preparation (boiled or filtered) and/or serving (from decaffeinated to Italian coffee), the average daily caffeine intake is in the range of 210–238 mg/person/day for US and Canada [4]. The causes for this increased demand and consumption pleasure result from the pharmacokinetic and pharmacodynamics properties of caffeine. After ingestion, caffeine is rapidly absorbed in the gastrointestinal tract, more precisely in the small intestine, reaching a high concentration in the blood after 30–60 min, with individual variations up to 120 min in cases with delayed gastric emptying [5]. A particularity of caffeine compared to other nutritive principles is the negligible pre-systemic metabolism which occurs in the liver, resulting in high absorption rates [6]. In addition, being a hydrophobic molecule, caffeine easily crosses the blood–brain barrier (BBB) and reaches the central nervous system (CNS), where it is also found in high concentrations [7]. At the CNS level, the substance exerts its neuro-stimulating action, increasing the degree of alertness and reducing fatigue, leading to better performance in psychomotor tasks requiring fast reactions [8]. These consequences and changes in healthy individuals have undoubtedly been demonstrated in multiple studies, both older [9] and more recent [10]. However, these cognitive benefits appear to result mainly because of the relief from withdrawal symptoms, rather than from a direct improvement in cognitive function [11]. The dose of caffeine remains the main determinant for the caffeine effects in the CNS, the locomotion activity being enhanced by low concentrations [12], while high caffeine concentrations have rather anxiogenic-like effects [13].

The effects of caffeine are not limited only to the CNS level, as there are relevant data also related to other peripheral organs and specific disorders. Thus, once the analgesic effect of caffeine was discovered, it was studied from the perspective of a pain modulator. A recent meta-analysis shows the benefit of adding at least 100 mg of caffeine to a standard dose of commonly used analgesics, resulting in a small but significant increase in the proportion of participants who experience a good level of pain relief [14]. Additionally, in the treatment of headache, the outcome is improved in the case of tension-type headache and migraine by adding caffeine as adjunctive therapy to the usual administered analgesic medication [15]. In addition, caffeine influences the metabolic processes that take place during physical activity in the periphery, such as in the heart, skeletal muscle, or adipocyte; in addition to reducing the perception of effort [16], caffeine modulates also other mechanisms involved in the case of hypoxia that are still incompletely known [17]. The gastrointestinal tract, including the mucosa, the motor function of the gut wall, and the brain-gut axis are also influenced by caffeine, according to recent data [18]. 

Starting from the data obtained in physiological conditions, clinical trials have also addressed the issue of the effects of caffeine in conditions of neurological pathology, as adjuvants in the therapy of neurodegenerative pathologies, most studies existing for Alzheimer’s disease (AD) and Parkinson’s disease. AD is the most common cause of dementia in the elderly, currently affecting over 50 million people worldwide [19], with estimates showing an increase in the incidence and prevalence of this disease in the coming decades [20]. Although first described by Alois Alzheimer over a century ago and with a lot of research already been done in order to develop a curative treatment, there is currently no effective therapeutic approach able to limit the occurrence of AD or slow the progression of the disease. The characterization and suppression of the most relevant risk factors for AD onset and evolution have also been important research directions, simultaneously with the extensive study of potential protective factors. Among the most studied protective factors with strong evidence for reducing the risk of AD we mention cognitive reserves [21], exercise [22], and administration of hormone therapy during the peri- and postmenopausal period [23]. Moreover, also other factors such as Mediterranean diet [24], coffee intake [25], and use of non-steroidal anti-inflammatory drugs (NSAID) [26] showed weak evidence and often contradictory results related to their influence on AD onset and evolution.

In this context of the lack of a precise direction regarding the proven benefit of caffeine in the etiopathogenesis of AD, this article aims to clarify this highly discussed topic by primarily making an extensive overview of the most important clinical studies performed on animal models and on the human subject. Without being a systematic review, after presenting the most relevant data available up to date from randomized-clinical trials (RCTs), the authors present the currently accepted theories that support the effectiveness of caffeine as a protective factor in AD evolution. Thus, outlining future research directions in both neuroengineering and the development of medicinal or nutritional protective factors, there is hope to develop an approach able to slow down the evolution of this disease.

## 2. Differentiation between Coffee and Caffeine and the Bias Issue 

**Figure 1:**
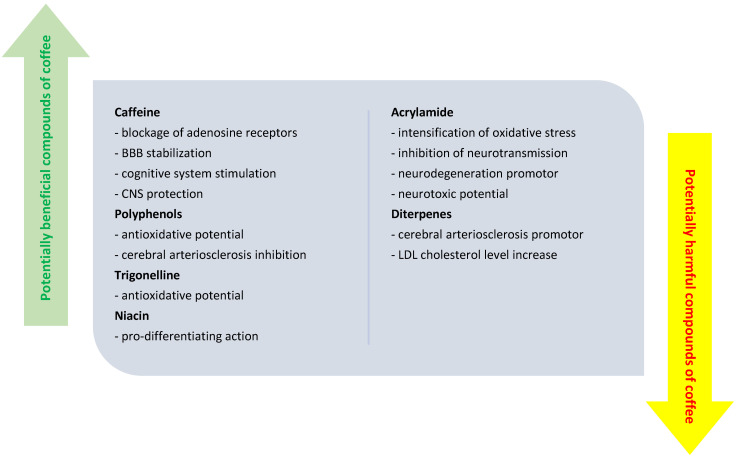
Biocomponents of coffee and their (potential) influence on different physiological processes at the central nervous system level.

A first element of interest that explains at least partially the heterogeneity of the results from the studies carried out so far is the weak differentiation between coffee consumption and caffeine administration, especially in clinical trials on humans. It is well known that coffee contains in addition to its main component, caffeine, also other substances with various biological roles, such as polyphenol, trigonelline, niacin, diterpenes, and acrylamide [27]. While some of the compounds such as caffeine, polyphenols, or trigonellines are suspected to have a neuroprotective effect, in terms of diterpenes and acrylamide, they are potentially harmful bioactive components, which support neurodegeneration rather than neuronal protection [28]. Figure 1 highlights the most important bio components of coffee and their likely effects at the CNS level. Or, for a maximum correctness of the results and the decrease of the bias, it is necessary that the studies (both the observational ones, and especially the interventional ones) to evaluate separately the roles of each biocomponent in the coffee. Thus, in this review, only the effects of caffeine will be illustrated; the effects of the other bioactive compounds remain to be studied in the near future. Furthermore, only studies that specify exactly the amount, frequency, and duration of caffeine administration were included in this review, as these parameters are highly relevant when studying biological effects of different nutrients.

## 3. Caffeine and the Correlation with Alzheimer’s Disease

Starting from preliminary epidemiological correlations or only theoretical data that supported the link between caffeine consumption and AD, researchers first conducted studies in silico, in vitro, and on experimental animal models in order to confirm a correlation of direct proportionality between the two concepts.

### 3.1. In Silico and In Vitro Studies

Despite the scarcity of research based on these kinds of approaches, data from available in silico studies have suggested that caffeine’s protective role in AD is related to the direct effect on amyloid beta peptide [29]. Molecular studies have shown the potential impact of caffeine in destabilizing the structure of amyloid beta complexes via multiple mechanisms, including the destruction of the inter-protein hydrogen bonds and the inhibition of amyloid beta oligomerization [29]. In addition, caffeine was also proposed as a potential inhibitor of acetylcholinesterase [30], improving the deficient cholinergic system encountered in AD patients.

In vitro cell culture studies rely on a different approach, as they try to elucidate some of the intracellular mechanisms that might explain the role of caffeine within AD pathology. Ca^2+^ [31], ryanodine, and NMDA receptors [32] are some of the most relevant molecules involved and potential therapeutic targets for the near future. 

### 3.2. Animal Model Studies

Different animal models were used, transgenic mice being the most popular ones. However, expending the research also in non-transgenic mice, rabbits, or nematodes, AD models brought supplementary evidence regarding the neuro-protective effects of caffeine assessed in preclinical studies (Table 1).

Among the transgenic mice models, the APPswe model was one of the first used. Initially described by Hsiao et al. [48], this line of mice expresses up to 6 times the human APP95 gene, resulting in an increased Aβ production in the animal’s brain. Senile plaques begin to form starting with 6–7 months of age, at 12 months of age being already in significant numbers in the cortex and hippocampus [49]. The model replicates other elements of AD-specific pathophysiology such as amyloidosis, vascular angiopathy, oxidative stress, and neuroinflammation. However, it remains an incomplete AD model, as the mouse does not develop neurofibrillary tangles, and neuronal and synaptic degeneration does not occur even in the context of the accumulation of large amounts of Aβ aggregates. Despite these obvious limitations, the model has been used in several studies that have shown the acute and long-term effects of caffeine on the memory deficit, a common finding in AD, as well as on other elements of anatomy and pathophysiology specific to the disease. Thus, even a single caffeine dose has been shown to have beneficial effects, stimulating a decrease in plasma and interstitial fluid Aβ levels [36]. However, in order to achieve neuroprotective effects, a chronic, long-term administration is required. Several studies have used various chronic regimens (from daily or even twice a day administration for periods up to 5 months), all of which have shown various degrees of beneficial effects, at least on some of the potentially neuroprotective mechanisms. For example, Zeitlin et al. [33] demonstrated the antiapoptotic and neuroprotective function of caffeine primarily through the modulation of signal transduction pathways, such as protein kinase cAMP-dependent (PKA), cAMP response element-binding protein (CREB), and c-Jun N-terminal kinase (JNK) pathways. According to the results of the study conducted by Han et al. [35], the protective role of caffeine against memory impairment was highlighted by improving spatial learning ability and memory capability, the molecular mechanisms underlying this evolution being increased expression of hippocampal brain-derived neurotrophic factor (BDNF) and tropomyosin receptor kinase B (TrkB). The neuroprotective effect can also be explained by the decrease in Aβ concentration in the hippocampus and based on the restoration of brain adenosine levels [34].

Another murine model intensely used in the study of AD is the THY-Tau22 model. Along with the accumulation of Aβ, another essential pathological marker for AD is the formation of neurofibrillary tangles, insoluble aggregates of Tau protein. This line of mice develops with age Tau-related neuropathological changes, initially Tau hyperphosphorylation, and later aggregates of Tau protein that simulate neurofibrillary tangles encountered in the cortex of humans with AD. Age is the main factor influencing the concentration of Tau in the mouse brain. If at 3 months the mild generation of hyperphosphorylated Tau begins, only at 6 months do the first cognitive impairments appear in the form of non-spatial memory impairment and appetitive responding [50]. Starting from 9 months of age, Tau neurofibrillary load is severe, and at 12 months, the loss of cells in the CA1 region begins. The mice do not exhibit changes in overall motor activity and do not have gross motor deficits [51]. Regarding the clinical trials in which this murine model was used in the study of the influence of caffeine on the CNS, they are few in number, in this review being highlighted only two of the most relevant ones. A first study led by Laurent et al. [37] is in line with the results mentioned before. Chronic caffeine administration for 10 months in THY-Tau22 male mice has been linked to a reduction in neuroinflammation and oxidative stress, and improvement of memory performance, especially spatial memory deficits. On the other hand, a recent study by Zappettini et al. [38] suggests that chronic coffee consumption during pregnancy is a risk factor for early AD in offspring.

The last mouse model discussed here is the triple transgenic mouse model (3xTg), a widely-used model in which neuropathological changes include both plaques and tangles. The first extracellular accumulations of Aβ are apparent by six months in the frontal cortex, at which time hyperphosphorilated Tau is still not observed. Later, at the age of 12 months, the senile plaques are much more extensive, along with obvious pathological accumulations of Tau protein. Other neuropathological elements such as synaptic dysfunction are also observable, even before the appearance of plaques or tangles, while cognitive impairments begin to manifest early, at 4 months, as a deficit in long-term retention [52]. Baeta-Corral et al. [39] studied the effects of chronic caffeine administration on a batch of 3xTg male mice, focusing on behavioral and psychological symptoms. According to the results, the possible beneficial effects of chronic caffeine administration such as improvement of learning and memory are covered by less favorable effects due to the aggravation of behavioral and psychological symptoms of dementia, and even by the appearance of neophobias.

Studies on non-transgenic mice represent another important part of research, being important for better understanding the physiological mechanisms and the possible effects of caffeine on cognition. Thus, in the case of C57BL/6N male mice, Badshah et al. [40] showed that administration of caffeine for 6 weeks will decrease LPS-induced oxidative stress, neuroinflammatory status and synaptic dysfunctions. Among the pathophysiological mechanisms involved, it is of interest to increase the expression of Nrf2, HO-1, and Bcl-2 simultaneously with the reduction of the expression of TLR-4, p-JNK, BAX, caspase-3, TNF-, COX-2, and NOS-2. Similar protective results have been reported in Fischer-344 young male rats treated with LPS, where caffeine decreases neuroinflammation by reducing the number of activated microglial cells [45]. The other mouse model in which caffeine studies have been performed is the adult CF1 male mouse model. The study conducted by Dall’Igna et al. [41] suggested the protective effect of caffeine, which by blocking A2AR prevents neurodegeneration and brain destruction.

The studies on animal models were not limited to mice only, as research also included various lines of rats. For example, studies have been performed on adult male Sprague–Dawley rats with accelerated aging [42], Wistar rats [44], and Fisher-344 male rats [45], the conclusions being in favor of the protective effect of caffeine on specific changes in AD rat models. Thus, chronically administered caffeine has antioxidant activity, by reducing 8-oxoguanine and NO levels [42]. Neuroprotection is also explained by anti-inflammatory activity, manifested mainly by reducing high levels of TNF-α and NF-κB pathways in the hippocampus and striatum [43,44]. Last but not least, by regulating the glutamate pathway, the activity of microglia is reduced [45], subsequently contributing to the reduction of cellular apoptosis and neurodegeneration.

Moreover, extensive research conducted also in other animal models brings further evidence to the protective effect of caffeine on neurodegeneration. In rabbit models, such as the New Zealand white (NZW) rabbit, Chen et al. [46] demonstrated the protective effect of chronic caffeine administration on the blood–brain barrier integrity, and also on other cellular phenomena observed in AD mouse and rat models, such as reduction in astrocytes and microglia activation. Finally, we mention also one study on a nematode model for AD, where caffeine administration was reported to prevent Aβ-induced toxicity and to delay the paralysis progression in worms [47]. 

However, it must be acknowledged that these models are not totally faithful copies of the cognitive changes encountered in patients with AD, as they can only partially replicate the anatomopathological changes [53]. Thus, the different murine models (double-transgenic [54] and triple-transgenic [55] AD mouse model) replicate the appearance of senile plaques formed by amyloid beta and hyperphosphorylated tau neurofibrils, the main characteristics of AD neurodegeneration, but with the abovementioned limitations.

The behavioral aspect of AD is also poorly replicated by these transgenic mouse models. As one of the first and most relevant symptoms in AD in humans is language impairments (from simple difficulties in remembering words to progressive primary aphasia), it must be said that animal models generally do not offer the possibility of testing this feature. Even in humans, currently valid cognitive tests are limited (mini-mental status examination, Montreal Cognitive Assessment test), and in transgenic mice, assessment is limited to spatial learning/memory, recognition/identification, exploratory behavior, and sensorimotor skills examination, providing only a little insight into the cognitive deficit that occurs in AD.

A major drawback for all the aforementioned models is the fact that they reflect more accurately the etiopathogenesis of the sporadic form of AD, or early-onset AD, which represents only a small percentage (5%) of all AD cases [56]. In addition, the translation of research from the field of basic studies into clinical trials on the human subject did not have the expected satisfactory results [57]. This explains the need to further develop more accurate models for AD in order to understand and explain the mechanisms of the disease hitherto unknown and for effective intervention measures for animal models to be successfully translated into humans.

### 3.3. Research in Humans

The encouraging results obtained from the studies conducted on animal models stimulated also extensive research on human subjects, both in terms of epidemiological and Mendelian randomization (MR) studies, the main conclusions being summarized in Table 2.

The first epidemiological studies on humans related to this topic date back to 2002, with preliminary encouraging results. According to Maia and de Mendonça [61], data from a small 54-case-control study suggested that a significant lower risk of AD is correlated with caffeine intake, independently of other variables such as age, sex, smoking, alcohol consumption, diabetes, and hypertension. This finding is in line with the data obtained after caffeine administration in patients with scopolamine-induced memory impairments [65]. Similar results were obtained also in the cohort-study conducted by Eskelinen et al. [62], where midlife daily coffee was associated with a decreased risk of AD in elderly patients. These positive results were only partially validated in larger and more recent studies. Of interest is the study of Gelber et al. [63], being the only research that found an association between higher caffeine intake and a decreased risk of developing AD neuropathological lesions. Regarding the protective feature of caffeine for cognitive impairment and dementia incidence, no significant association could be made. Finally, the work of Larsson and Wolk [64] additionally questioned the beneficial role of caffeine in dementia, as no association was found between coffee intake and AD incidence. 

Regarding the MR studies, we first mention the works of Kwok et al. [58], which does not point in a clear direction regarding the coffee consumption and AD risk. Newer MR studies conducted by Larsson et al. [59] and Zhang et al. [60] are suggestive for the negative impact of coffee consumption on AD development. Conducted on large populations, the results are in opposition to that expected based on earlier observational studies. It is worth mentioning however that the MR research did not study the effect of caffeine solely on AD risk, but the association of coffee and AD, the bias risks already being discussed before in this work. Similarly, heterogenous results were also obtained when the effect of coffee was studied on another significant neurodegenerative disease, multiple sclerosis (MS). While MS may have common pathophysiological processes such as neuroinflammation and oxidation with AD, coffee’s effects on MS onset and progression are still a matter of debate [66,67]. 

## 4. The Pathophysiological Basis of Caffeine Effects in Alzheimer’s Disease

The abovementioned studies conducted on animal models have suggested various possible mechanisms which may explain caffeine’s effect in AD. The most frequently proposed hypotheses are related to the reduction of oxidative stress [37,40,44] and neuroinflammation [37,42,45], although other potential pathophysiological mechanisms such as caffeine’s antiapoptotic effect [33] or the inhibition of astrocyte activation [46] are also taken into consideration. Regarding research in humans, the antioxidant effect of caffeine has been the main argument in studies that have shown a protective effect of coffee related to dementia [61,62].

In order to better understand the effect of caffeine in AD, it should be noticed that the main way in which caffeine obtained from regular coffee consumption performs its functions at the CNS level is by blocking the A1 and the A2A adenosine receptors. The most important among adenosine receptors is A1 receptor (A1R), a glycoprotein containing 326 amino acids, having the highest affinity and being mostly expressed at CNS level, in the cortex, thalamus, cerebellum and also other CNS structures. The A2A receptor (A2AR) is distributed mainly in the dopaminergic areas of the CNS, such as striatum globus pallidus, nucleus accumbens, olfactory tubercle, bulbus olfactorius, and nucleus nervi acustici [68]. All adenosine receptors are members of the G protein-coupled receptor (GPCR) family, with A1R belonging to the Gi family, while A2AR being a member of Gs family. Thus, activation of A1R in the presynaptic membrane will inhibit adenylyl cyclase activity, reduce the concentration of cyclic adenosine-3,5 monophosphate cAMP and, via inositol 1,4,5-triphosphate IP3, reduce Ca^2+^ influx, subsequently reducing the excitability of nerve conduction [69]. A1R exerts its neuroprotective role also in postsynaptic neurons, by hyperpolarizing the membrane and reducing neuronal excitability. When A2AR is activated, protein kinase A (PKA) pathway is activated, interfering with nuclear factors-activated-κB (NF-κB) and regulating gene expression (Jain et al., 2020) [70]. Indeed, Zeitlin et al. [33] demonstrated that caffeine stimulates PKA activity in APPswe, thus explaining its neuroprotective effect. Subsequently, unlike A1R, A2AR activation will promote release of excitatory neurotransmitters. By activating the mitogen-activated protein kinases (MAPK), A2AR can increase the production of collagen, inhibit the peroxidation of neutrophils, and, at the blood vessel level, mediate vasodilatation. 

In AD, according to the adenosine receptor balance theory proposed by Yan et al. [71], the decrease in A1R expression happens simultaneously with the increase in A2AR expression, leading to disruption in inhibition and excitation processes and, eventually, to cognitive dysfunction. Strong evidence suggests overexpression of A2AR also during ageing at cortical and hippocampal levels in rat models [72]. In studies conducted on humans, the blockade of A2AR by selective competitors leads to a normalization of synaptic and cognitive dysfunctions [73]. Via the blockade of A2AR, caffeine protects against synaptic toxicity of Aβ, but also against non-neuronal cells that are involved in AD pathogenesis. Neuroinflammation is an essential step in AD onset and development, with astrocyte and microglia activation playing central roles in promoting and sustaining the inflammatory process. A2AR activation modulates both astrocyte and microglia, with harmful effects also for surrounding neurons. Activated microglia, by producing several inflammatory cytokines and neurotoxic factors, is the key player in neuroinflammation [74,75]. In this context, multiple evidence [76,77,78] showed that by blocking A2AR, direct inactivation of microglia occurs, subsequently controlling neuroinflammation. Reduction of inflammation and microglia inactivation after caffeine administration were already found in several AD rat models [42,44]. A2AR also mediates the glutamate pathway, downregulating the neurotransmitter release and uptake [79], diminishing the noxious effects of direct calcium entry into the neurons and the inflammatory reactivity of microglia. Apoptosis is also indirectly inhibited via A2AR-glutamate inactivation, as pro-apoptotic markers caspase 3 and cytochrome C were modulated by A2AR antagonists, as research on a model of ischemia reperfusion suggested [80]. 

A second mechanism of interest for caffeine is the upregulation of the expression of nuclear factor erythroid 2-related factor 2 (Nrf-2). Nrf-2, a basic region leucine zipper (bZip) transcription factor, has become an interesting molecule for researchers, as studies conducted during the last decade showed its role in resistance to oxidative stress. Nrf2 knockout mice demonstrated an increased susceptibility to the development of several pathological conditions associated with oxidative pathology [81]. In order to exerts its anti-oxidative functions, Nrf2 regulates several genes involved in oxidative metabolism. Two main genes are of interest and related to caffeine, namely the genes coding hem oxygenase-1 (HO-1) and superoxide dismutase (SOD1), two substrates that play an important role in glutathione degradation, such as glutathione S-transferase and glutathione cysteine ligase catalytic subunit (GCLC) [82]. The oxidative stress theory as one pivotal point in neurodegeneration has been extensively discussed and remains even today a viable target for therapeutic approaches [83]. In addition to Nrf2, other reactive oxygen and nitrogen species and regulating factors are involved in the complex cascade of pathological oxidation, and the influence of caffeine related to this intricate pathway is still to be fully determined. 

**Figure 2:**
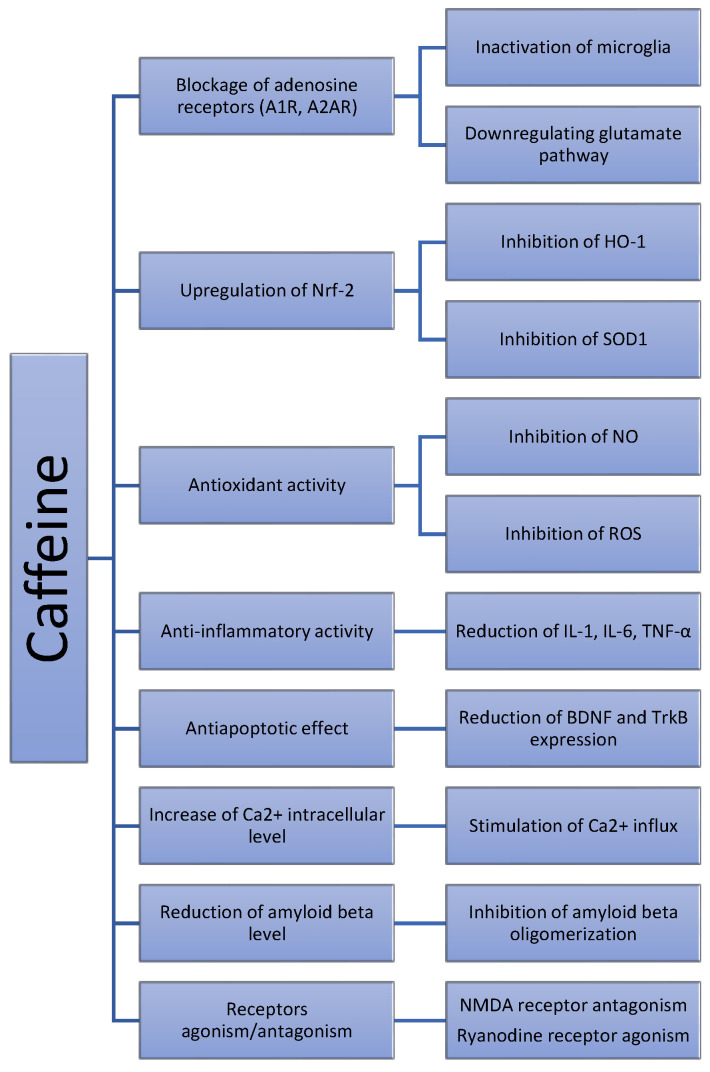
The influence of caffeine in neurodegeneration—proposed pathophysiological mechanisms.

Finally, other incompletely studied molecular pathways could also be involved (see Figure 2), as in vitro and in silico studies suggest. For example, the role of Ca^2+^ in the regulation of different pathophysiological mechanisms related to AD is currently well known [84], research suggesting that caffeine enhances intracellular Ca^2+^ levels via both direct and indirect mechanisms [31]. On the other hand, hypotheses such as the direct effect of caffeine on amyloid beta oligomers were only evident in experimental studies [29], not being validated in animal models [47].

## 5. Impact of Computational Modeling on Caffeine—Alzheimer’s Disease Association

Although studies on large cohorts of human subjects have not shown a protective effect of coffee on the onset of AD, no definitive conclusion can be drawn yet. Neuroengineering has become increasingly helpful in understanding the pathogenesis of AD and in studying the effects of different drugs or biologically active compounds in pathological conditions [85]. Computational models represent a very heterogeneous group, ranging from single cell and biochemical models, to increasingly complex representations such as system level models [86]. Given the multiple etiopathogenic theories of AD, amyloid, Tau, cholinergic, and oxidative stress hypotheses being the most studied ones, there is currently no theoretical model that accurately simulates AD, all models having their limitations. Most of the earlier biochemical models focused on the influence of amyloid beta accumulation, now becoming more complex by incorporating other variables such as neuro-inflammation, mitochondrial dysfunction, lipid metabolism dysregulation, or the role of ApoE [87]. Single cell models, on the other hand, focus mainly on the study of ion channels modulation, membrane potential, and synaptic transmission alterations, with the latest models simulating the behavior of hippocampal CA1 and CA3 neurons in beta-amyloid accumulation, characterizing short-term plasticity alterations, synapse disfunctions, and firing probability [88]. As AD alters several neuronal networks (mainly in the hippocampus, but also in other regions of interest of the CNS such as basal ganglia and the thalamus), systemic computational approaches tend to offer more relevant insights on functional modifications, trying to explain and forecast memory loss, impaired learning ability, and behavioral changes, common symptoms encountered in AD patients.

Nowadays, there is no computational model which simulates the effects of caffeine on AD pathogenesis. With numerous approaches available, choosing the most suitable model remains debatable. As the potential protective effect of caffeine is thought to be related mainly to modulation of oxidative stress and inflammation, one good option would be the use of a biochemical model focusing on neuroinflammation. However, with growing knowledge on neuronal receptors with high caffeine affinity, the use of an updated single cell model could eventually reveal the whole signaling pathway, offering new targets for future antidementia drugs. Finally, the use of a systemic computational model (preferably including several neural circuits affected by AD) mimics most accurately the clinical impairments of AD patients, offering insights on the consequences of caffeine intake on memory and other cognitive abilities impaired in dementia.

There is thereby an essential need for improving current existing computational models and developing others based also on neuroinflammation and oxidative stress in addition to beta-amyloid pathology. Moreover, as coffee also contains its main component, caffeine, there should be an almost equal interest for conducting computational analysis on other compounds with potential neuroprotective impacts, such as polyphenols or trigonellines [89]. There is a clear need to address AD-related issues also from a computational perspective; mathematical models, although imperfect, bringing additional arguments for further clinical trials on human subjects, especially in cases where studies to date have provided contradictory conclusions.

## 6. Conclusions and Future Research Directions

Based on theoretical considerations and fundamental research conducted on several animal models, coffee, through its main component caffeine, seemed to act in a protective manner in AD. However, further larger cohort studies in humans did not reproduce the favorable results, most recent results suggesting an increased risk of AD associated with increased coffee consumption. As previously mentioned, the substrate complexity of coffee and the lack of clinical trials on controlled caffeine long term administration in humans makes this topic still open for debate, the possible utility of computational models worth to be considered.

The proposed pathophysiological pathways, which explain caffeine effects in the complex AD ethiopathogenesis, are important targets for developing future effective antidementia drugs. Reducing oxidative stress by targeting key molecules such as the adenosine receptors or the Nrf-2 related pathway can offer adjuvant therapeutic resources in addition to the ones currently used in clinical practice. Further studies are also important in order to fully explain cellular and molecular factors related to AD, AD risk, and protective factors. Finally, it is mandatory also to develop new nano-molecules capable of reproducing with a higher efficacy the effects of caffeine at the CNS level by facilitating BBB crossing and the affinity to the receptors involved in molecular pathways also relevant to AD pathogenesis.

## Figures and Tables

**Table 1:** Acute and long-term caffeine intake effects in different AD animal models.

Animal Model	Advantages of the Model	Drawbacks of the Model	Study Design	Main Results/Findings	Reference
APPswe mouse model	High concentration of Aβ even in young model (starting with 6–7 months of age) Replication of amyloidosis, vascular angiopathy, oxidative stress, and neuroinflammation	Absence of neurofibrillary tangles, no global neuronal or synaptic losses, no clear abnormalities in the brain structures associated with learning and memory	Administration of 1.5 mg p.o. caffeine for 2 weeks, every 12 h in 9.5-month-old mice in order to investigate the effects of caffeine on the signal transduction pathways in cognitively important areas of the mouse brain	Neuroprotective and antiapoptotic effect by stimulating PKA activity Increasing the level of phosphorylated CREB Decreasing JNK and ERK phosphorylation	Zeitlin et al., 2011 [33]
			Administration of 0.3 g/L p.o. caffeine in drinking water for 5.5 months, starting with 4-month-old mice in order to determine the neuroprotective effects of long-term dietary caffeine intake	Protective effect against cognitive impairment Reduction in Abeta levels in the hippocampus, restoration of brain adenosine levels No effect on A1R and A2AR hippocampal density and expression in the cerebral cortex and hippocampus	Arendash et al., 2006 [34]
			Administration of 0.75 mg/day or 1.5 mg/day p.o. of caffeine for 8 weeks in 12-month-old mice in order to investigate the effects of caffeine intake on the memory deficits, BDNF and TrkB expression	Increasing in spatial learning ability and memory capability Increasing in the expression of hippocampal BDNF and TrkB. Protective role against memory impairment	Han et al., 2013 [35]
			Acute administration regimen (single administration of 1.5 mg i.p. caffeine in 3- to 4-month-old; single administration of 1.5 mg i.p. or p.o. caffeine in 14-month-old) Long-term regimen (1.5 mg p.o. caffeine twice-daily for 7 days in 15- to 20-month-old; caffeine: 1.5 mg p.o. caffeine in two administrations on one day every 4th day for 2 months, 20-month-old)	Improvement of cognitive functions after long-term caffeine intake Reduction of Aβ interstitial fluid level after acute caffeine administration, but no effect on Aβ elimination Decreased Aβ plasma levels after single dose and chronic administration Reduction of soluble Aβ cortex and hippocampus level and insoluble Aβ hippocampus level after chronic caffeine administration	Cao et al., 2009 [36]
THY-Tau22 mouse model	Simulation of neurofibrillary tangle formation and pathological influence in AD Age-dependent neuropathological changes, which offer the possibility of study in different stages of the disease	No Aβ/senile plaques cerebral load	Administration of 0.3 g/L p.o. caffeine in drinking water for 10 months in 2-month-old male mice in order to study the effect of chronic caffeine intake on the development of hippocampal tau protein pathologies and spatial memory disorders	Prevention of spatial memory deficits Improvement of memory performance Reduction of neuroinflammation and decrease in the hippocampal level of hyperphosphorylated tau protein. Reduction of oxidative stress (reduced expression of MnSOD and EAAT3)	Laurent et al., 2014 [37]
			Chronic administration of 0.3 g/L p.o. caffeine in drinking water in female mice, starting of administration 2 weeks before mating and ending at 15th postnatal day in order to evaluate the effects of long-term caffeine exposure during pregnancy in offspring	Induction of physiological disorders and accelerated cognitive disorders Potential risk factor for early stages of AD.	Zappettini et al., 2019 [38]
3xTg mouse model	Neuropathological changes include both plaques and tangles Extracellular Aβ deposits are apparent as earlier as by six months in the frontal cortex Translates functional deficits such as synaptic dysfunction and LTP deficits	Tau pathology evident by 12 months	Chronic administration of 0.3 mg/mL caffeine in drinking water p.o. for 7 months, starting with 6-month-old male mice to investigate the effects of long-term caffeine administration on memory and learning	Reduction of motor activity, total horizontal activity, and emotionality in the behavioral tests Increasing of spontaneous motor activity (to a greater extent at night) Aggravation of BPSD-like behaviors, anxiety-related behaviors, or neophobia adversely affected possible beneficial effects	Baeta-Corral et al., 2018 [39]
C57BL/6N mouse	Most used breed in clinical studies Different modifications possible (lipopolysaccharide—LPS, genetics)	More susceptible to morphine addiction, atherosclerosis, and age-related hearing loss	Chronic caffeine administration of 30 mg/kg/day i.p. for 6 weeks in C57BL/6N male mice treated with LPS in order to examine caffeine effect on LPS-induced oxidative stress, neuroinflammation, apoptotic cell death, neurodegeneration, and synaptic impairment	Reduction of LPS-induced oxidative stress, neuroinflammation, and synaptic dysfunctions	Badshah et al., 2019 [40]
Adult CF1 male mice	Multipurpose model Suited for safety and efficacy testing		Single and chronic administration of caffeine in order to assess its effect on cognitive impairment in AD induced CF1 mouse model by i.c.v. A25–35 administration	Prevention of cognitive impairment, neurodegeneration, and brain destruction	Dall’Igna et al., 2007 [41]
Adult male Sprague–Dawley rats with accelerated aging	Multipurpose model Calmness Ease of handling Fast growing	Increased (and very variable) rate of tumor growth	Chronic caffeine administration (3 mg/kg/day i.p. for 60 days) impact on neurodegeneration induced by D-galactose-aging rat model	Reduction of oxidative stress, neuroinflammation, neuronal cell apoptosis, neurodegeneration, synaptic dysfunction and memory deficits	Ullah et al., 2015 [42]
			Chronic administration of instant decaffeinated coffee (p.o.) at 120 or 240 mg/kg for 2 weeks	Inhibition of scopolamine-induced memory impairment Suppression of TNF-α and NF-κB pathway at hippocampus level	Jang et al., 2013 [43]
Adult male Wistar rats	One of the most popular rat models used worldwide (first rat model) More active than other rat models High survival rate	Very high spontaneous incidences of foci of altered hepatocytes (FAH) Affected by vascular tumors	Chronic caffeine administration (20 mg/kg i.p. for 30 days) in adult male Wistar rats treated with AlCl3 (100 mg/kg p.o. for 30 days)	Antioxidant and anticholinesterase activity against AlCl3-induced neurotoxicity Reduction of oxidative stress parameters (NO level) Decrease of AChE and Na+/K+-ATPase activity in the cerebral cortex and hippocampus Anti-inflammatory properties—reduction of TNF-α levels in the hippocampus and striatum	Hosny et al., 2019 [44]
Fischer-344 male rats	Excellent model for aging research Extensive research (more than 5 decades) in carcinogenicity studies	High prevalence of severe nephropathy at advanced ages	Chronic administration of caffeine for 2 or 4 weeks to young rats and for 2 weeks to aged rats in order to assess caffeine effect on neuroinflammation	Potential protective effect against LPS-induced neuroinflammation	Brothers et al., 2010 [45]
New Zealand white rabbit cholesterol-induced AD model	Preferred in laboratory testing because of their docility and good health Small size, easy and low-cost maintenance, high availability		Chronic caffeine administration (3 mg/day in 50 mL of drinking water for 12 weeks) in order to investigate the effects on blood–brain barrier leakage in rabbits fed with cholesterol-enriched diet	Prevention of BBB dysfunction Reduction of astrocytes activation Reduction in microglia density	Chen et al., 2008 [46]
*Caenorhabditis* *elegans* (nematode model)	Possesses homologs of about two-thirds of all human disease genes Useful model for aging research Ease of maintenance	Lack of certain anatomical structures of mammals (BBB, blood transport system) Lack of long-range transcriptional regulation	Administration of 10% coffee extract (3.6 mM caffeine) in the agar medium in order to assess the effects of caffeine on the Aβ-induced toxicity in *Caenorhabditis elegans*	Prevention of Aβ-induced toxicity Delay in the paralysis progression No reduction in Aβ expression, Aβ aggregation or distribution	Dostal et al., 2010 [47]

**Table 2:** Caffeine/coffee and Alzheimer’s disease—most relevant studies in humans.

Study Methodology	Study Population Cases vs. (Controls)	Main Conclusion	Reference
Mendelian randomization studies			
Two-sample MR Summary-level data Study cohort—International Genomics of Alzheimer’s Project	17,008 (37,154)	No evidence supporting a causal relationship between coffee and AD (no beneficial effect)	Kwok et al., 2016 [58]
Two-sample MR MR Egger regression Summary-level data Study cohort—International Genomics of Alzheimer’s Project	17,008 (37,154)	Suggestive association between coffee genetic score and increased risk of AD (*p* = 0.01)	Larsson et al., 2017 [59]
Two-sample MR Summary-level data Study cohort—publicly available databases (two genome-wide association studies)	54,126 (375,833)	Genetically predicted coffee consumption may be associated with an increased risk of AD (*p* < 0.05)	Zhang et al., 2021 [60]
Epidemiological studies			
Case-control study	54 (54)	Caffeine intake (daily during the 20 years preceding AD diagnosis) was associated with a significantly lower risk for AD, independently of other possible confounding variables	Maia and de Mendonça, 2002 [61]
Cohort study	48 (1409)	Coffee drinking (3–5 cups daily) at midlife was associated with a decreased risk of AD later in life	Eskelinen et al., 2009 [62]
Case-control study	118 (3494)	Caffeine intake in midlife was not associated with cognitive impairment or dementia Higher caffeine intake was associated with lower odds of having neuropathological lesion at autopsy	Gelber et al., 2011 [63]
Cohort study	1299 (28,775)	No association between coffee consumption and AD incidence	Larsson and Wolk, 2018 [64]

## Data Availability

All data and materials supporting the results of the present study are available in the published article.
